# A case of pilomatrix carcinoma found in the shoulder

**DOI:** 10.1093/jscr/rjaf574

**Published:** 2025-08-04

**Authors:** Yunan Han, Abid Qureshi, Dhipthika Srinivasan, Ahmad Attal, Atakan Isik, Yingxian Liu, Luca Milone

**Affiliations:** Department of Surgery, The Brooklyn Hospital Center, 121 DeKalb Avenue, Brooklyn, NY 11201, United States; Department of Surgery, The Brooklyn Hospital Center, 121 DeKalb Avenue, Brooklyn, NY 11201, United States; Department of Surgery, The Brooklyn Hospital Center, 121 DeKalb Avenue, Brooklyn, NY 11201, United States; St George’s University School of Medicine, Department of Surgery, The Brooklyn Hospital Center, 121 DeKalb Avenue, Brooklyn, NY 11201, United States; Department of Surgery, The Brooklyn Hospital Center, 121 DeKalb Avenue, Brooklyn, NY 11201, United States; Department of Pathology, The Brooklyn Hospital Center, 121 DeKalb Avenue, Brooklyn, NY 11201, United States; Department of Surgery, The Brooklyn Hospital Center, 121 DeKalb Avenue, Brooklyn, NY 11201, United States

**Keywords:** pilomatrix carcinoma, pilomatrixoma, malignancy, surgical excision

## Abstract

To date, ~200 cases of pilomatrix carcinoma have been reported in the literature. These tumors usually present as atypical, solitary, firm nodules, most commonly in the head and neck region. Published data indicate that these tumors exhibit aggressive behavior and have a high potential for recurrence. Therefore, surgical excision with wide margins is recommended. We report a case of pilomatrix carcinoma located on the shoulder, initially presenting as a small pimple with purulent discharge in a 64-year-old man. We also review the relevant literature, with particular focus on the clinical characteristics, early identification, and treatment modalities associated with this disease.

## Introduction

Pilomatrix carcinoma (PC) is an extremely rare malignant skin tumor arising from the uncontrolled proliferation of follicular matrix cells. First reported by Lopansri and Mihm in 1980 as “calcifying epitheliocarcinoma of Malherbe” or ``pilomatrixoma carcinoma'' [1]. PC has remained poorly understood, with only 205 reported cases in 118 studies between 1980 and 2024 [1, 2]. It typically presents as a solitary, firm, atypical nodule, most commonly on the head and neck [2–4].

The pathogenesis is unclear, but proposed mechanisms include either de novo development or malignant transformation from pilomatrixoma, a benign counterpart [5]. While pilomatrixoma typically affect children and young adults (with a slight female predominance), PC occurs more often in older males (median age 58 years; 70.4% male) [2, 6]. Both pilomatrixoma and PC share CTNNB1 gene mutations, supporting a “two-hit hypothesis”: an initial mutation results in β-catenin accumulation leading to pilomatrixoma, and a second mutation affects tumor suppressor or proto-oncogenes, resulting in malignant transformation [7].

Due to its rarity, there is no standardized treatment protocol for PC. Wide local excision with clear margins is the current treatment of choice, though re-excision may be needed because of the tumor's aggressive behavior and high recurrence rate. Mohs micrographic surgery may offer better margin control and lower recurrence [1, 8]. Given its atypical presentation, aggressive behavior, and potential for recurrence, local invasion, and metastasis, early recognition and appropriate surgical intervention are crucial for optimal patient outcomes. Here, we present a case of PC on the shoulder, initially deemed to be squamous cell carcinoma.

## Case report

**Figure 1:**
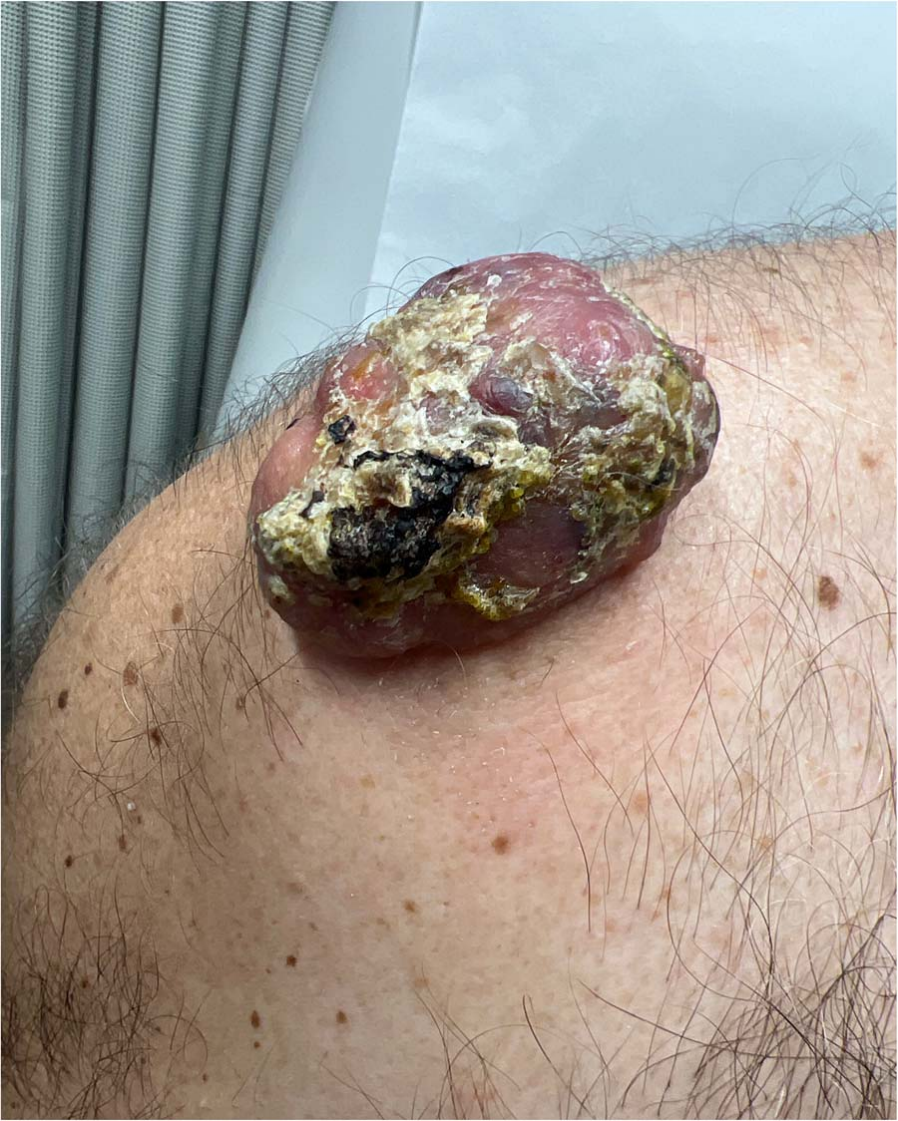
Ulcerated and lobular mass seen on right shoulder during initial consultation.

A 64-year-old male presented with a right shoulder mass that had progressively grown over 1 year. Patient reported that the lesion began as a small pimple that would rupture and drain purulent discharge. Over the last few months, drainage ceased, though the lesion caused discomfort. His past medical history was remarkable was hypertension, diabetes, coronary artery disease, and extensive tobacco use. Physical examination revealed a 4 × 4 cm purplish, ulcerated, globular mass on the proximal right shoulder without drainage (Fig. 1). Ultrasound showed a 4 × 2.5 × 3.9 cm heterogeneous hypoechoic mass with cystic foci in the soft tissues. The decision was made to take the patient to the operating room, we performed excision of right shoulder mass.

**Figure 2:**
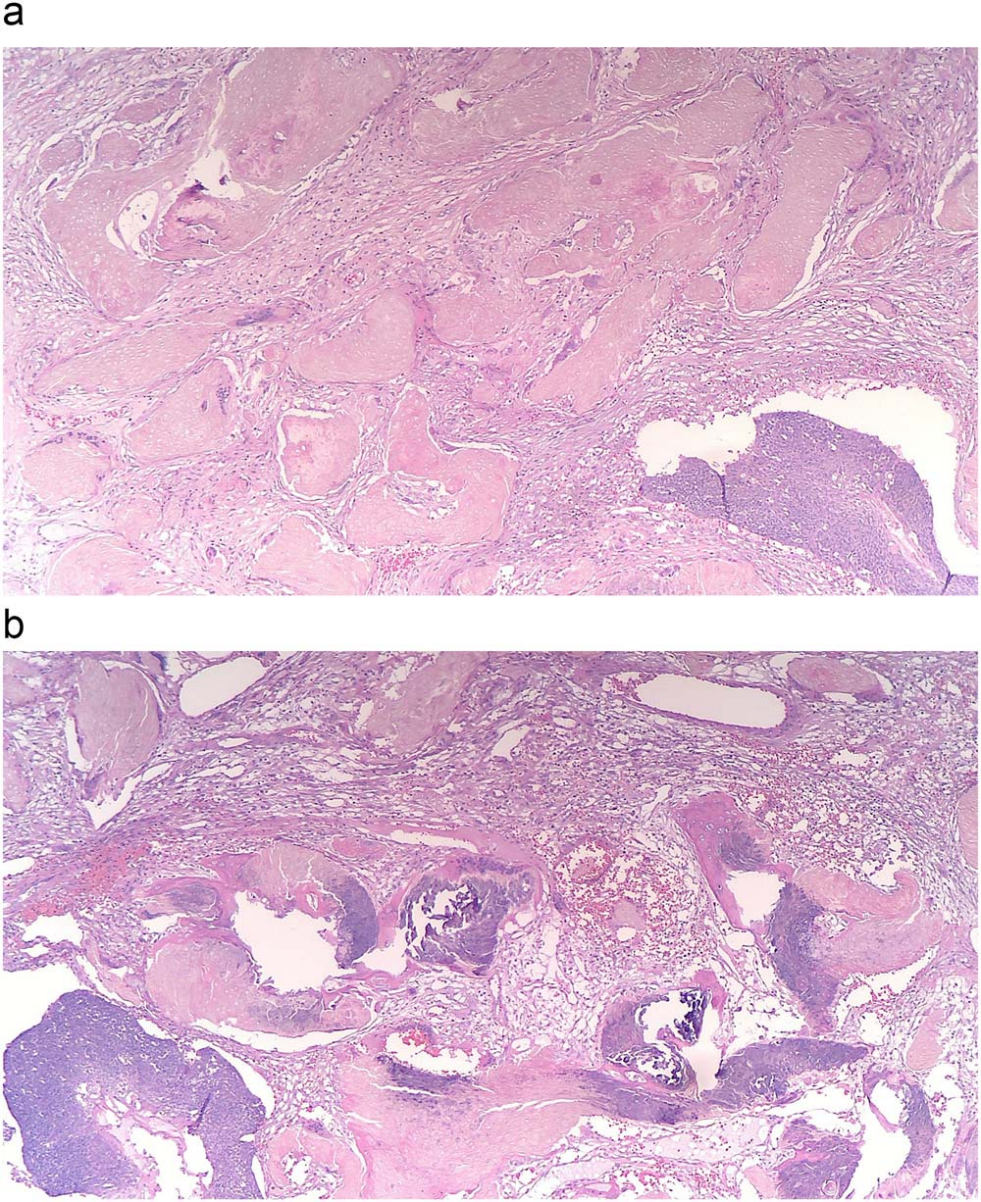
(a and b) Histopathology shows nests of central necrosis, abrupt keratinization, anucleated ghost cells, and microcalcification, consistent with a pilomatrixoma background.

**Figure 3:**
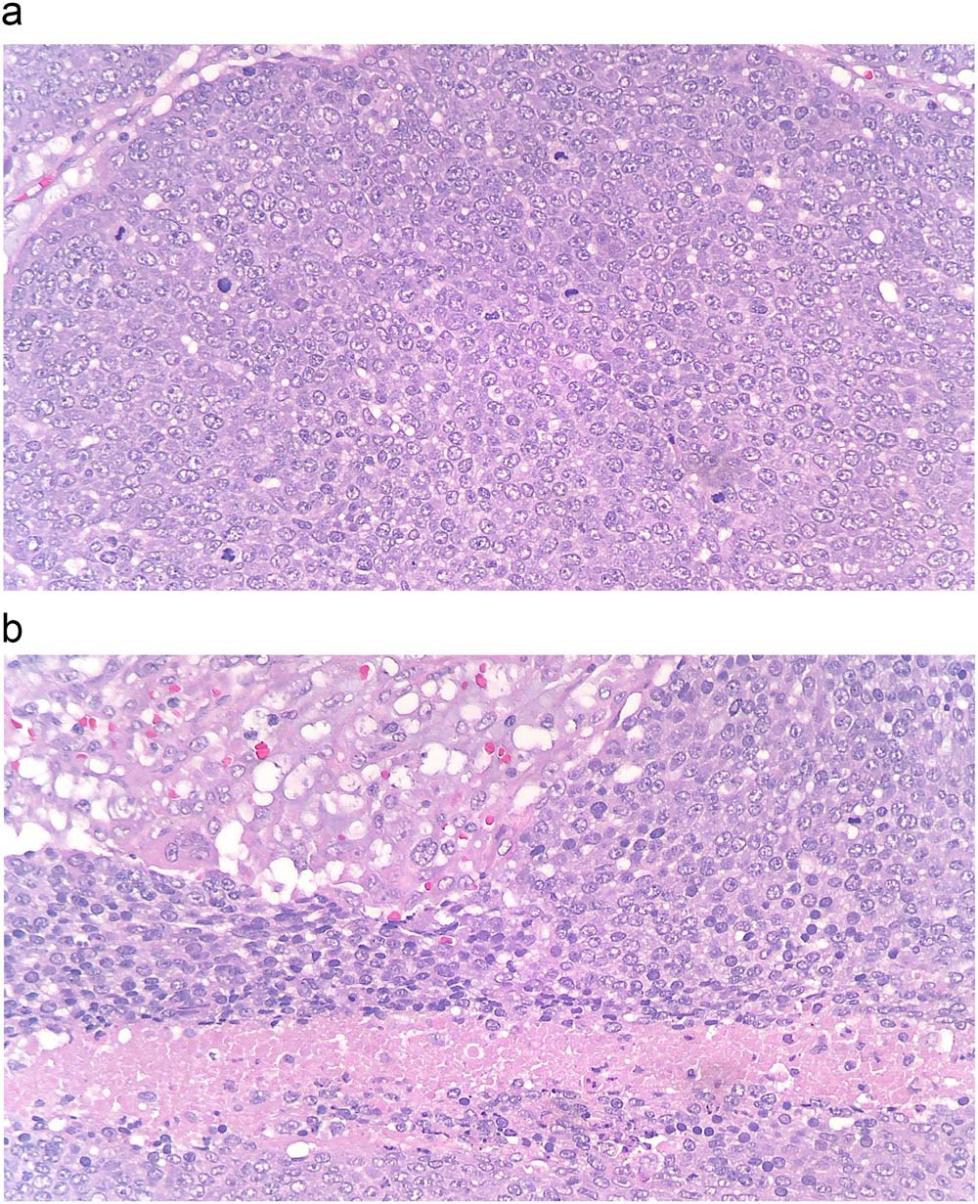
(a and b) At the periphery of the tumor, nests of basoloid and squamoid cells showed marked cytologic atypia with enlarged irregular nuclei, prominent nucleoli, increased mitotic figures, and foci of necrosis.

Pathology of the mass was initially read as poorly differentiated basaloid squamous cell carcinoma with necrosis and calcifications. Histopathology demonstrated nests of central necrosis, abrupt keratinization, anucleated ghost cells, and microcalcification (Fig. 2a and b). At the periphery of the tumor, nests of basoloid and squamoid cells showed marked cytologic atypia with enlarged irregular nuclei, prominent nucleoli, increased mitotic figures, and foci of necrosis (Fig. 3a and b). Immunohistochemical stains reveal that carcinoma cells are positive for p63, p40, and ki-67 (40%). Due to the uncertainty in diagnosis, the specimen was sent to a tertiary center for confirmation, which resulted with the presence of “shadow cells” indicating PC with basaloid and squamoid features. All margins were negative for carcinoma. Positron emission tomography-computed tomography was conducted, showing minimal activity in the region of skin and underlying subcutaneous infiltration in the right shoulder region favored to be related to evolving post-surgical inflammation. As the tumor was 4 cm, Grade 3 disease, with 2.5 cm depth of invasion, per National Comprehensive Cancer Network guidelines the criteria was met for Stage 3 disease. Patient was started on radiation treatment of the right shoulder with scheduled nodal basin surveillance to assess for recurrence. There were no postoperative complications.

## Discussion

While literature on PC is limited, several consistent clinical features are recognized. PC primarily affects older men (3:1 male-to-female ratio) and may develop either de novo or from pre-existing pilomatrixoma. Over half of PC cases occur on the head and neck, and around 16% metastasize to lymph nodes, lungs, or bone, though most remain localized [7–9].

Both PC and pilomatrixoma involve mutations in the CTNNB1 gene, leading to β-catenin accumulation. Normally, Exon 3 of this gene targets β-catenin for degradation through phosphorylation in the WNT signaling pathway. Mutation disrupts this regulation, allowing nuclear accumulation of β-catenin and activation of transcription factors involved in follicular differentiation and hair cell development [6, 10, 11].

Histologically, PC is characterized by irregular nests of large, anaplastic basaloid cells with hyperchromatic nuclei, prominent nucleoli, and numerous mitotic figures. Central areas of the tumor often show necrotic debris and ghost cells. Additional findings may include ulceration, vascular invasion, and infiltrative growth patterns [11]. These features distinguish PC from benign pilomatrixoma and other cutaneous malignancies.

Clinically, PC usually presents as a firm, painless, solitary mass. Violaceous discoloration and ulceration are commonly seen but non-specific, making clinical diagnosis difficult. PC can resemble basal cell carcinoma, dermal cysts, or melanoma. Its non-specific presentation often leads to misdiagnosis or delayed recognition. Rapid tumor growth, rather than size alone, is a more reliable indicator of malignancy and should prompt biopsy [6, 9, 10].

Treatment of PC depends on the extent of the malignancy. In research, the majority of cases have been treated with surgery, chemotherapy, and radiation either as adjuvants or primary treatments. Surgical resection with wide margins is generally recommended to maintain local involvement of the tumor. Although when it arises in sensitive areas such as the head and neck, Mohs micrographic surgery is indicated as this type of surgical resection has better cosmetic outcomes. In many cases, surgery is often followed by radiation or chemotherapy to prevent recurrence and metastasis. The role of adjuvant radiotherapy has not been fully studied, however, has provided adequate local tumor control and in cases where surgical resection is not possible, can be an alternative [8–12].

In conclusion, the identification and prognosis of PC is important with regards to early surgical resection and multidisciplinary consultations involving medical and radiation oncologists. PC should be considered on the differential list of hard solitary tumors of the head and neck region. Wide surgical resection remains the gold standard for treatment of PC, followed by adjuvant radiotherapy. Follow-up examinations of patients are warranted, such as full body skin examinations annually.

## Data Availability

Data gathered during this scoping review is available upon reasonable request to the corresponding author.
